# Propionic Acidemia‐Induced Proarrhythmic Electrophysiological Alterations in Human iPSC‐Derived Cardiomyocytes

**DOI:** 10.1002/jimd.70030

**Published:** 2025-04-29

**Authors:** Anabel Cámara‐Checa, Mar Álvarez, Josu Rapún, Sara Pérez‐Martín, Roberto Núñez‐Fernández, Marcos Rubio‐Alarcón, Teresa Crespo‐García, Lourdes R. Desviat, Eva Delpón, Ricardo Caballero, Eva Richard

**Affiliations:** ^1^ Department of Pharmacology and Toxicology, School of Medicine Universidad Complutense de Madrid Madrid Spain; ^2^ Instituto de Investigación Gregorio Marañón Madrid Spain; ^3^ Centro de Investigación Biomédica en Red de Enfermedades Cardiovasculares (CIBERCV) Instituto de Salud Carlos III (ISCIII) Madrid Spain; ^4^ Centro de Biología Molecular Severo Ochoa UAM‐CSIC Universidad Autónoma de Madrid Madrid Spain; ^5^ Instituto Universitario de Biología Molecular (IUBM) Madrid Spain; ^6^ Instituto de Investigación Sanitaria Hospital La Paz (IdiPaz) Madrid Spain; ^7^ Centro de Investigación Biomédica en Red de Enfermedades Raras (CIBERER) Instituto de Salud Carlos III (ISCIII) Madrid Spain

**Keywords:** heart, human‐induced pluripotent stem cell‐derived cardiomyocytes, ion currents, patch‐clamp, propionic acidemia

## Abstract

Propionic acidemia (PA) is a metabolic disorder caused by a deficiency of the mitochondrial enzyme propionyl‐CoA carboxylase (PCC) due to mutations in the *PCCA* or *PCCB* genes, which encode the two PCC subunits. PA may lead to several types of cardiomyopathy and has been linked to cardiac electrical abnormalities such as QT interval prolongation, life‐threatening arrhythmias, and sudden cardiac death. To gain insights into the mechanisms underlying PA‐induced proarrhythmia, we recorded action potentials (APs) and ion currents using whole‐cell patch‐clamp in ventricular‐like induced pluripotent stem cells‐derived cardiomyocytes (hiPSC‐CMs) from a PA patient carrying two pathogenic mutations in the *PCCA* gene (p.Cys616_Val633del and p.Gly477Glufs*9) (PCCA cells) and from a healthy subject (healthy cells). In cells driven at 1 Hz, PCC deficiency increased the latency and prolonged the AP duration (APD) measured at 20% of repolarization, without modifying resting membrane potential or AP amplitude. Moreover, delayed afterdepolarizations appeared at the end of the repolarization phase in unstimulated and paced PCCA cells. PCC deficiency significantly reduced peak sodium current (*I*_Na_) but increased the late *I*_Na_ (*I*_NaL_) component. In addition, L‐type Ca^2+^ current (*I*_CaL_) density was reduced, while the inward and outward density of the Na^+^/Ca^2+^ exchanger current (*I*_NCX_) was increased in PCCA cells compared to healthy ones. In conclusion, our results demonstrate that at the cellular level, PCC deficiency can modify the ion currents controlling cardiac excitability, APD, and intracellular Ca^2+^ handling, increasing the risk of arrhythmias independently of the progressive late‐onset cardiomyopathy induced by PA disease.

## 1 Introduction

Cardiac complications are increasingly recognized as critical factors affecting morbidity and mortality in patients with some inherited metabolic diseases [1]. These disorders, which disrupt normal metabolism due to enzyme deficiencies or malfunctions in metabolic pathways, can lead to systemic effects that include profound impacts on cardiac function. The intense metabolic activity of the heart makes it especially vulnerable to disturbances in metabolic homeostasis, which can lead to a range of cardiac manifestations, from structural changes to functional impairments [2].

In this context, propionic acidemia (PA, OMIM606054, ORPHA: 35), a rare autosomal recessive metabolic disorder, emerges as a poignant example of how inherited metabolic anomalies can precipitate severe cardiac complications. PA results from mutations in the *PCCA* or *PCCB* genes that encode the two subunits of the propionyl‐CoA carboxylase protein (PCC, E.C.6.4.1.3), a crucial enzyme in the catabolism of several amino acids (isoleucine, valine, threonine, and methionine), odd‐chain fatty acids, and cholesterol. PCC catalyzes the carboxylation of propionyl‐CoA to D‐methylmalonyl‐CoA, and its deficiency leads to the accumulation of propionyl‐CoA and other metabolites, such as 3‐hydroxypropionic acid, 2‐methylcitric acid, propionyl‐CoA, propionylcarnitine, and propionic acid [3]. These accumulated compounds are toxic and/or inhibit diverse metabolic pathways, resulting in hyperglycinemia, hyperammonemia, ketosis, and lactic acidosis [4]. As a result, PA manifests as a multi‐systemic chronic disease, mainly affecting energy‐demanding organs such as the brain, muscle, and heart [4].

Cardiac symptoms have been recognized as important complications of PA since 1993 [1, 5, 6]. However, they do not appear to be related to the severity of the disease [7, 8], and are considered progressive late‐onset complications and one of the major causes of mortality associated with PA [9]. Previous studies have outlined the presence of cardiomyopathy potentially presenting in various forms including dilated (DCM) [10], hypertrophic (HCM) [11], or left ventricular noncompaction cardiomyopathies (LVNC) [12]. Additionally, electrophysiological disturbances have been observed, notably QT prolongation, which may be associated with cardiac rhythm alterations, including ventricular ectopic beats or couplets, polymorphic ventricular tachycardia, and potentially fatal arrhythmias [1, 13, 14, 15]. Dietary protein restriction and other treatments, like liver transplantation, can improve cardiac complications in PA patients, although some may still develop cardiac abnormalities despite these interventions [16]. Therefore, further exploration of alternative therapeutic approaches is needed, as current treatments, while effective in some cases, are not sufficient to address all the symptoms associated with the disease.

The precise mechanism through which PA leads to heart disease remains unclear. Multiple interrelated processes, including mitochondrial dysfunction, oxidative stress, and changes in gene expression mediated by microRNAs (miRNAs) and/or epigenetic modifications, have been explored as contributing factors to PA cardiac dysfunction in cellular and animal models [17, 18, 19]. Additionally, compromised Ca^2+^ handling, as observed in a hypomorphic PA mouse model, has been identified as a potential pathogenic mechanism linked to cardiac dysfunction and ventricular arrhythmias in PA [20].

The hypomorphic PA mouse model has significantly advanced our understanding of PA physiopathology, particularly regarding cardiac aspects [19, 20]. However, due to physiological differences between mouse and human hearts, such as heart rate and calcium handling, this model has its limitations [21, 22]. The challenge of obtaining human ventricular myocytes has led to the development of new patient‐specific models, among which human induced pluripotent stem cell‐derived cardiomyocytes (hiPSC‐CMs) stand out [23]. These cells provide a valuable platform for studying disease mechanisms within a relevant human context. To gain deeper insight into the mechanisms underlying the electrophysiological alterations associated with PA, we recorded action potentials (APs) and ion currents in ventricular‐like hiPSC‐CMs derived from a PA patient carrying two pathogenic variants in the *PCCA* gene (p.Cys616_Val633del and p.Gly477Glufs*9) and in hiPSC‐CMs from a healthy individual. The findings reveal that PCC deficiency can severely disrupt some of the ionic mechanisms that control cardiac excitability, AP duration (APD), and cytosolic Ca^2+^ handling at the cellular level, thus heightening the risk of arrhythmias independently of the late‐onset cardiomyopathy progression characteristic of the disease.

## 2 Materials and Methods

### 2.1 Maintenance of hiPSC Lines

The iPSC lines used in this work were: (i) a *PCCA*‐deficient iPSC line (PCCA23‐FiPS4F6 or UAMi001‐A) generated by reprogramming of patient‐derived fibroblasts with defects in the *PCCA* gene (c.1899+4_1899+7delAGTA; p.(Cys616_Val633del) and c.1430‐?_1643+?del; p.(Gly477Glufs*9)) using Sendai virus [24]; and (ii) a healthy control iPSC line (FIPS Ctrl2‐SV4F‐1) obtained from Banco Nacional de Líneas Celulares del Instituto de Salud Carlos III (ISCIII, Madrid, Spain). The pluripotent characteristics of the *PCCA*‐deficient iPSC line were analyzed through the expression of key pluripotent genes by RT‐qPCR. Additionally, the expression of transcription factors OCT4, NANOG, and SOX2, along with surface markers SSEA3, SSEA4, TRA‐1‐60, and TRA‐1‐81, was assessed using immunocytochemistry and flow cytometry. Furthermore, the cells demonstrated the ability to form derivatives of all three germ layers (endoderm, mesoderm, and ectoderm) upon embryoid body differentiation and maintained a normal karyotype [24].

Human iPSC lines were maintained on Matrigel‐coated (hESC‐qualified matrix, Corning, New York, NY, USA) tissue culture dishes 60 mm with mTESR Plus medium (StemCell Technologies, Vancouver, BC, Canada) with regular medium changed every other day. iPSCs passaging was performed every 4 days using ReleSR (StemCell Technologies) and 10 μM Rock inhibitor (StemCell Technologies) at a 1:3–1:5 splitting ratio.

### 2.2 Differentiation of iPSCs Into Cardiomyocytes

iPSCs maintained in mTESR Plus medium were dissociated into single cells using StemPro Accutase (Gibco, Waltham, MA, USA). 1 × 10^6^ cells in 1.5 mL of mTESR Plus medium supplemented with 10 μM Rock inhibitor were seeded onto matrigel‐coated 12‐well plates. Cardiomyocyte differentiation was carried out using STEMdiff Cardiomyocyte Differentiation and Maintenance Kits (StemCell Technologies) according to the manufacturer's instructions. Cardiomyocyte characterization was performed through the expression analysis of several cardiac‐specific markers, such as cardiac troponin T (cTnT), α‐smooth muscle actin, GATA4, and α‐actinin 2 by immunocytochemistry, as described [25]. The efficiency of generating cTnT‐positive cardiomyocytes in both cell lines, as assessed by flow cytometry, was similar (≈95%) [25].

### 2.3 Cardiomyocyte Purification

Cardiomyocyte isolation was performed after 25–30 days of cardiomyocyte differentiation from iPSCs, using a PSC‐derived cardiomyocyte isolation kit (Miltenyi Biotec, Bergisch Gladbach, Germany) with several modifications. Briefly, cells from one well of a 12‐well plate were washed twice with 1X PBS, then harvested using the STEMdiff Cardiomyocyte Dissociation Kit (StemCell Technologies). After centrifugation at 200×g for 5 min the cell pellet was resuspended in 1 mL of Buffer A, containing 1× PBS pH 7.2, 0.5% bovine serum albumin, and 2 mM EDTA. The cell suspension was centrifuged at 300×g for 5 min, and the resulting pellet was resuspended in 80 μL of Buffer A. 20 μL of a non‐cardiomyocyte depletion cocktail was added to the cell suspension. Following a 5‐min incubation, the cells were washed by adding 1 mL of Buffer A and centrifuged at 200×g for 5 min. The cell pellet was then resuspended in 80 μL of Buffer A and incubated with 20 μL of anti‐biotin microbeads for 10 min. Magnetic separation was subsequently performed according to the manufacturer's instructions using LS columns and pre‐separation filters (70 μm) (both from Miltenyi Biotec), and an appropriate MACS separator. After a final centrifugation at 200×g for 5 min, the enriched cardiomyocytes were resuspended in STEMdiff Cardiomyocyte Support Medium (StemCell Technologies) containing 10 μM Rock inhibitor and seeded onto Matrigel (Corning Matrigel Basement Membrane Matrix)‐coated 24‐well plates with a cover slip (12 mm diameter, Epredia, DA Breda, Netherlands). The culture medium was replaced with STEMdiff Cardiomyocyte Maintenance Kit (StemCell Technologies) the next day, and the medium was subsequently changed every two days over a period of 7–9 days.

### 2.4 Patch‐Clamp Recordings

The coverslips containing cultured cells were placed in a chamber mounted on the stage of an inverted microscope (Nikon TMS; Nikon Co. Tokyo, Japan). Cells were perfused at ≈1 mL/min with external solution (see the corresponding compositions below). APs and ion currents were recorded at room temperature (21°C–23°C) by means of the whole‐cell patch‐clamp technique using Axopatch‐200B patch‐clamp amplifiers and pCLAMP10.8 software (Molecular Devices, San José, CA, USA) [26, 27, 28, 29]. Recording pipettes were pulled from 1.0 mm o.d. borosilicate capillary tubes (GD1, Narishige Co. Ltd. Tokyo, Japan) using a programmable patch micropipette puller (Model P‐2000 Brown‐Flaming, Sutter Instruments Co., Novato, CA, USA) and were heat‐polished with a microforge (Model MF‐83, Narishige). Micropipette resistance was kept below 1.5 MΩ for the Na^+^ current (*I*_Na_) or between 3 and 7 MΩ for the L‐type Ca^2+^ current (*I*_CaL_), the Na^+^/Ca^2+^ exchanger current (*I*_NCX_), and APs when filled with the internal solution and immersed in the external solution. In all the experiments, series resistance was compensated manually using the series resistance compensation unit of the Axopatch amplifier, and ≥ 80% compensation was achieved. The remaining access resistance after compensation and cell capacitance were 1.7 ± 0.9 MΩ and 43.9 ± 6.9 pF (*n* = 25) for healthy hiPSC‐CMs. Therefore, no significant voltage errors (< 5 mV) due to series resistance were expected with the micropipettes used under our experimental conditions. Capacitance was not significantly modified by the PCC deficiency (53.7 ± 6.8 pF, *n* = 24; *p* > 0.05). To minimize the contribution of time‐dependent shifts of channel availability during I_Na_ recordings, all data were collected 5–10 min after establishing the whole‐cell configuration [26, 27]. Recordings of APs and currents were sampled at 50 (*I*_Na_), 10 (APs) or 2 (*I*_CaL_ and *I*_NCX_) kHz, filtered at 10 (*I*_Na_) or at 1 (APs, *I*_CaL_ and *I*_NCX_) kHz, and stored on the hard disk of a computer for subsequent analysis. To minimize the influence of variability, experiments were performed in a large number of cells obtained from at least 3 different batches.

APs were recorded in hiPSC‐CMs using the current‐clamp configuration of the patch‐clamp technique in unstimulated cells or in cells driven at 1 Hz by applying depolarizing‐current pulses of 2 ms in duration at 1.5–2 times the threshold [26, 27]. The frequency of the APs generated by unstimulated cells and resting membrane potential (RMP), AP amplitude (APA), and AP duration measured at 20% (APD_20_), 50% (APD_50_), and 90% (APD_90_) of repolarization in cells driven at 1 Hz were measured. The external solution contained (in mM): NaCl 148, NaH_2_PO_4_ 0.4, MgCl_2_ 1, glucose 5.5, KCl 5.4, HEPES 15, and CaCl_2_ 1.8 (pH 7.4 with NaOH), whereas the internal solution contained (in mM): KCl 150, K_2_ATP 4.46, phosphocreatine 5, HEPES 5, EGTA 1, MgCl_2_ 1, and β‐hydroxybutyric acid 2 (pH 7.2 with KOH) [26, 27].

The external solution used to record I_Na_ contained (in mM): NaCl 20, CsCl 115, MgCl_2_ 1.5, nifedipine 1 μM, HEPES 5, CaCl_2_ 1, glucose 10 (pH 7.35 with CsOH) [26, 27]. Recording pipettes were filled with an internal solution that contained (in mM): NaF 10, CsF 110, EGTA 10, CsCl 20, and HEPES 10 (pH 7.35 with CsOH). To construct the current–voltage (I‐V) relationships for *I*_Na_, 50‐ms pulses in 5 mV increments from −120 mV to potentials between −80 and +50 mV were applied (in the corresponding figures, traces and density values for currents recorded up to +20 mV are shown). Peak I_Na_ amplitude was measured as the difference between the peak current and the current at the end of the pulse. The persistent or late *I*_Na_ (*I*_NaL_) was recorded by applying 500‐ms pulses from −120 to −30 mV, measured as the leak‐subtracted inward current remaining at the end of the pulse and represented as the ratio (in %) between late and peak *I*_Na_. In some experiments, the *I*_NaL_ was also recorded as the current sensitive to 1 μM tetrodotoxin (TTX) [27]. The external solution for *I*_CaL_ recordings contained (in mM): NaCl 137, CsCl 5.4, MgCl_2_ 1.25, HEPES 10, CaCl_2_ 1.2, and glucose 5.5 (pH 7.35 with NaOH). Recording pipettes were filled with an internal solution containing (in mM): CsCl 120, tetraethylammonium (TEA) Cl 20, MgCl_2_ 1, MgATP 5.2, HEPES 10, and EGTA 10 (pH 7.2 with CsOH). To record *I*_CaL_, 200‐ms pulses from −50 to +60 mV in 10‐mV steps were applied. *I*_CaL_ amplitude was measured as the difference between the peak current and the current at the end of the pulse. To quantify the *I*_Na_ and *I*_CaL_ activation and inactivation kinetics, a monoexponential function was fitted to the activation phase of maximum peak current traces (*I*_Na_) or to the traces generated by pulses to +10 mV (*I*_CaL_). The time course of inactivation was characterized by fitting a biexponential function to the decay phase of maximum peak *I*_Na_ traces or of *I*_CaL_ traces recorded at +10 mV, yielding the fast and slow time constants of inactivation (*τ*_finact_ and *τ*_sinact_).

Conductance–voltage curves of *I*_CaL_ and *I*_Na_ were constructed by plotting normalized conductance as a function of membrane potential. Conductance was estimated for each experiment with this equation:

$$G=I/\left(V_{m}-E_{\mathrm{rev}}\right)$$

where *G* is conductance at test potential (*V*m), *I* represents the peak maximum current at *V*_m_, and *E*_rev_ is the reversal potential. To determine *E*_rev_, current density–voltage relationships obtained in each experiment were fitted to a function of this form:

$$I=\left(V_{m}-E_{\mathrm{rev}}\right)\times G_{\max}\times \left(1+\exp\left[V_{m}-V_{h}\right]/k\right)-1$$

where *I* is the peak current elicited at test potential *V*_m_, *G*_max_ is maximum conductance, and *k* is the slope factor. A Boltzmann function was fitted to activation/conductance–voltage curves to obtain the midpoint (*V*_h_) and slope (*k*) values.

To record *I*_NCX_, the external solution contained (in mM): NaCl 140, CsCl 4, CaCl_2_ 2.5, MgCl_2_ 1.2, HEPES 5, glucose 10, glibenclamide (10 μM), nifedipine (1 μM), atropine (1 μM), and ouabain (10 μM) (pH 7.4 with NaOH). After obtaining control recordings (total current), the external solution was changed to another containing 5 mM Ni^2+^/0 Ca^2+^. *I*_NCX_ was measured as the Ni^2+^‐sensitive current and was obtained by digital subtraction of Ni^2+^‐insensitive current from total currents. The internal solution to record *I*_NCX_ contained (mM): CsCl 110, NaCl 20, HEPES 10, MgCl_2_ 0.4, glucose 5, TEA·Cl 20, EGTA 5, MgATP 4, and CaCl_2_ 1 (pH 7.2 with CsOH) [29]. The protocol to record I_NCX_ consisted of 300 ms‐pulses from −40 mV to potentials ranging between −80 and +80 mV. *I*_NCX_ was calculated as the difference between the current measured in the presence of extracellular Ca^2+^ and that measured in the presence of 5 mM Ni^2+^/0 Ca^2+^ [29]. I‐V curves of *I*_NCX_ were corrected according to a liquid junction potential of 15.7 mV.

In all cases, current amplitude was normalized by cell capacitance to obtain current density for each experiment.

### 2.5 Statistical Analysis

Results are expressed as mean ± SEM. Small‐sized samples were first analyzed to determine the distribution of the variables (normality) using the Shapiro–Wilk and Kilmogorov‐Smirnov tests. When these tests demonstrated a normal distribution, parametric tests were used. Unpaired *t* tests were used to assess statistical significance. When a statistically significant difference was determined by this test, a Pearson correlation was performed to confirm that the observed differences were not due to random sampling. To account for repeated sample assessments, data were analyzed using multilevel mixed‐effects models. Variance was similar between groups that were being statistically compared. A value of *p* < 0.05 was considered significant. For the different groups of experiments, sample size was chosen empirically according to previous experience in the calculation of experimental variability. No statistical method was used to predetermine sample size. The cellular experiments were not blinded due to the nature of the experimental design and platforms, but all the data were analyzed in an identical manner for all conditions to eliminate possible operator bias. Variations in the number of measurements were due to differences in sample availability and experimental conditions. We have accounted for these discrepancies in our analyses to ensure statistical robustness.

## 3 Results

In this study, we have used the iPSC line derived from a female patient with PA, currently 17 years old, who had an early‐onset presentation, with the following *PCCA* gene variants: (GRCh38.p14, NM_000282.4: c.1899+4_1899+7delAGTA; p.(Cys616_Val633del) and c.1430‐?_1643+?del; p.(Gly477Glufs*9)). This patient has experienced developmental delays, hypotonia, recurrent infections, and chronic pancreatitis. Notably, there is no evidence of cardiac abnormalities observed to date, although the patient has not undergone specific cardiological evaluations for the past few years.

### 3.1 PCC Deficiency Produces Alterations in APs Recorded in hiPSC‐CMs

**FIGURE 1:**
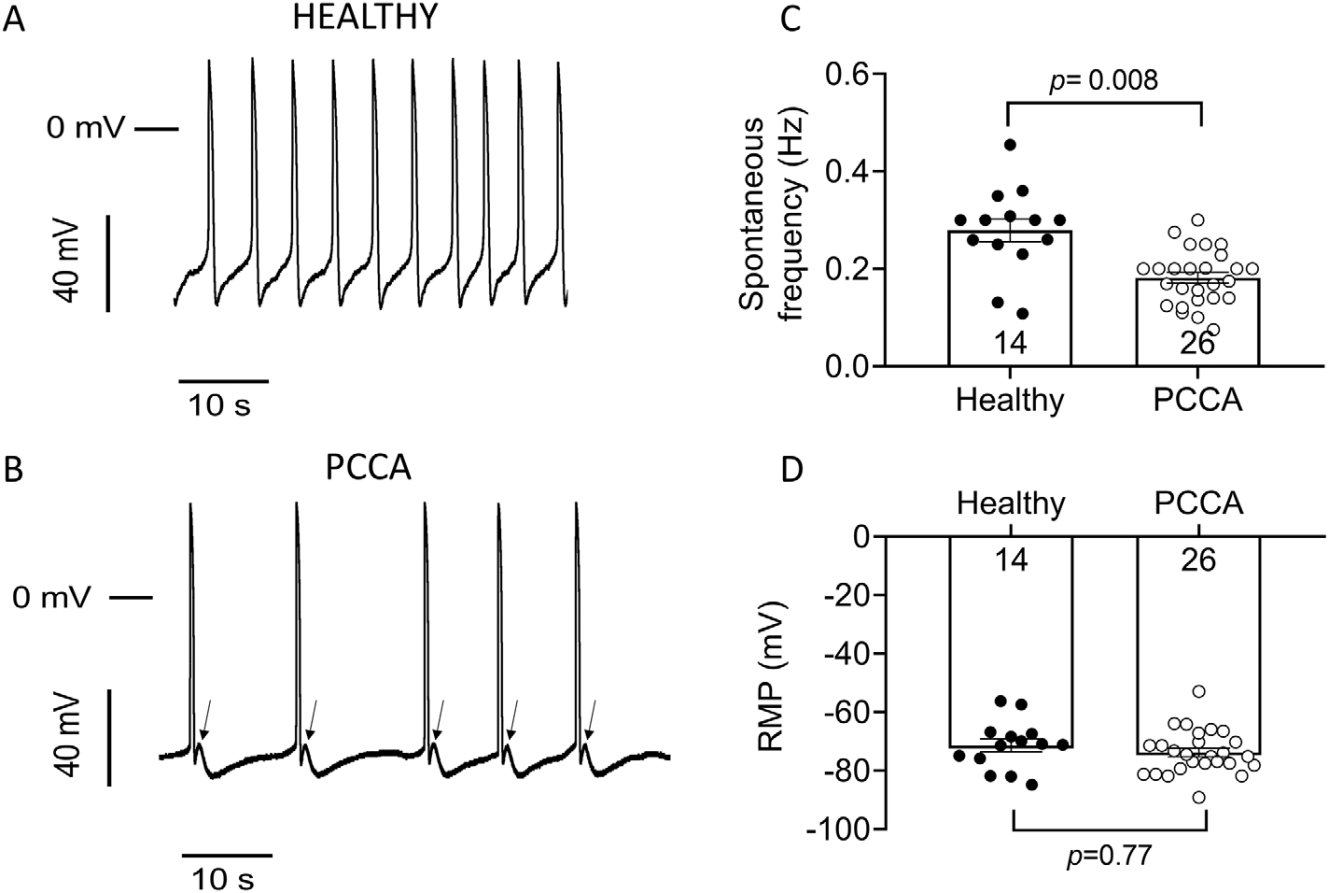
Characteristics of spontaneous APs recorded in hiPSC‐CMs. (A and B) Series of spontaneous APs generated by hiPSC‐CMs obtained from the healthy donor (A) or the *PCCA*‐deficient patient (B). In panel B, the appearance of delayed afterdepolarizations is indicated by arrows. (C, D) Mean values of spontaneous frequency (C) and RMP (D) measured in both experimental groups. In panels C and D, each bar represents the mean ± SEM of the “*n*” experiments/cells indicated in each corresponding figure, and each dot represents one experiment/cell. In C and D, the *p* values from Student's *t* comparisons are indicated.

We first investigated whether PCC deficiency produces alterations in the AP characteristics. To this end, APs were recorded in ventricular‐like hiPSC‐CMs differentiated from a healthy donor (Healthy cells) and from the *PCCA*‐deficient patient (PCCA cells). During the differentiation process, mixtures of spontaneously electrically active hiPSC‐CMs were obtained, including atrial‐, nodal‐, and ventricular‐like myocytes, although the differentiation procedure used in this study yielded a relatively homogeneous population of ventricular‐like cardiomyocytes. Nevertheless, in the AP comparisons, we discarded cells that did not meet the requirements for AP parameters typical of ventricular‐like myocytes. For example, only cells with APA larger than 80 mV, APD_90_ longer than 350 ms, and a repolarization fraction ([APD_90_–APD_50_]/APD_90_) smaller than 0.33, when paced at a frequency of stimulation of 1 Hz, were included [27]. Figure 1A,B, shows representative traces of a series of APs generated spontaneously by two different hiPSC‐CMs, differentiated from the healthy donor and the *PCCA*‐deficient patient, respectively. It is evident that the firing frequency of these automatic APs was smaller in PCCA than in healthy cells (*n* ≥ 14; *p* < 0.05) (Figure 1A–C), although the RMP was not significantly different (*p* > 0.05) (Figure 1D). Interestingly, APs recorded in PCCA cells exhibited a transient and small depolarization of the membrane potential once repolarization was finished (arrows in Figure 1B), which is suggestive of delayed afterdepolarizations (DADs).

**FIGURE 2:**
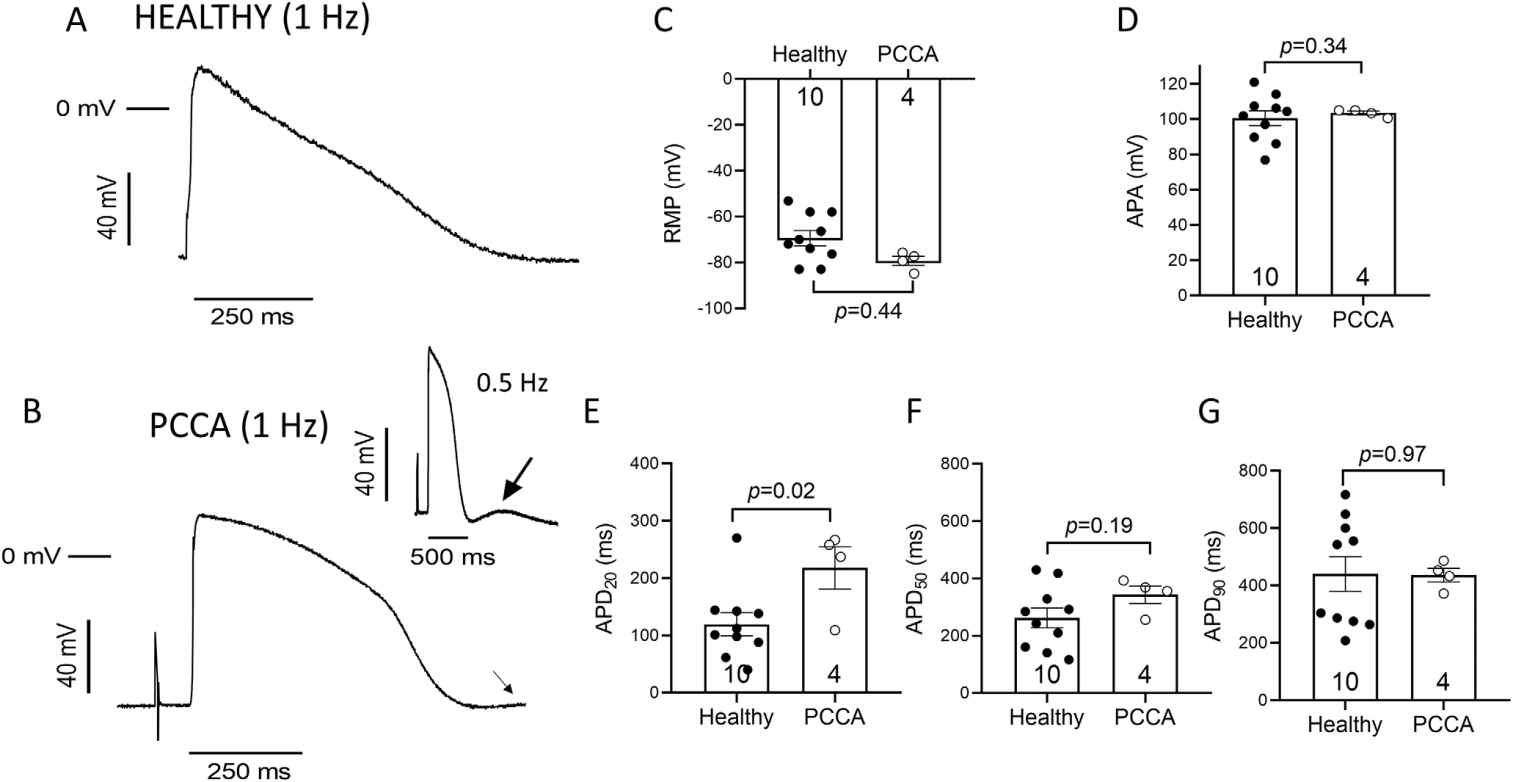
Characteristics of APs recorded in hiPSC‐CMs driven at 1 Hz. (A, B) Representative traces of APs generated by hiPSC‐CMs obtained from the healthy donor (A) or the *PCCA*‐deficient patient (B). The inset shows a trace of an AP generated by a cell driven at 0.5 Hz. In panel B, the inset, the respective transient and slow depolarization phases just after repolarization are highlighted by arrows. (C–G) Mean values of RMP (C), APA (D), APD_20_ (E), APD_50_ (F), and APD_90_ (G). In panels C–G, each bar represents the mean ± SEM of the “*n*” experiments/cells indicated in each corresponding figure and each dot represents one experiment/cell. In panels C–G, the *p* values from Student's *t* comparisons are indicated.

We next recorded APs in cells driven at 1 Hz. In the hiPSC‐CMs derived from the healthy donor, the application of the electrical stimulus instantaneously triggered the AP, which exhibits the characteristic morphology of APs generated by ventricular cardiomyocytes (Figure 2A). In hiPSC‐CMs derived from the patient, a large fraction of cells (≈85%) did not follow the pacing at 1 Hz, that is, these cells did not generate an AP after each stimulus. Moreover, in the cells that followed the pacing, the application of the stimulus did not instantly trigger the AP upstroke, but rather took a few tens of milliseconds (Figure 2B). These results suggest that cell excitability is greatly depressed in cardiomyocytes differentiated from the patient‐derived iPSCs, although neither the RMP nor APA were significantly altered (*n* ≥ 4, *p* > 0.05) (Figure 2C,D). In PCCA cells, the initial repolarization phase was slowed compared to healthy cells, leading to a significant increase in the APD_20_ (Figure 2E), with no differences in the APD_50_ or APD_90_ (Figure 2F,G). Moreover, as observed when recording the spontaneous activity, a slowly depolarizing phase (arrow in Figure 2B) at the end of repolarization was apparent in paced cells, suggesting the presence of DADs, although at 1 Hz they could not be completed before the arrival of the next stimulus (Figure 2B). Nevertheless, DADs were clearly visible in PCCA hiPSC‐CMs driven at 0.5 Hz (inset of Figure 2B).

All these results suggest that PCC deficiency reduces cardiac excitability, prolongs the duration of the early repolarization phase, and leads to the appearance of DADs. Based on these findings, and to gain mechanistic insights, we explored whether PCC deficiency affects the ion currents whose alterations could be responsible for these features [30].

### 3.2 PCC Deficiency Reduces Peak *I*_Na_ and Increases *I*_NaL_

**FIGURE 3:**
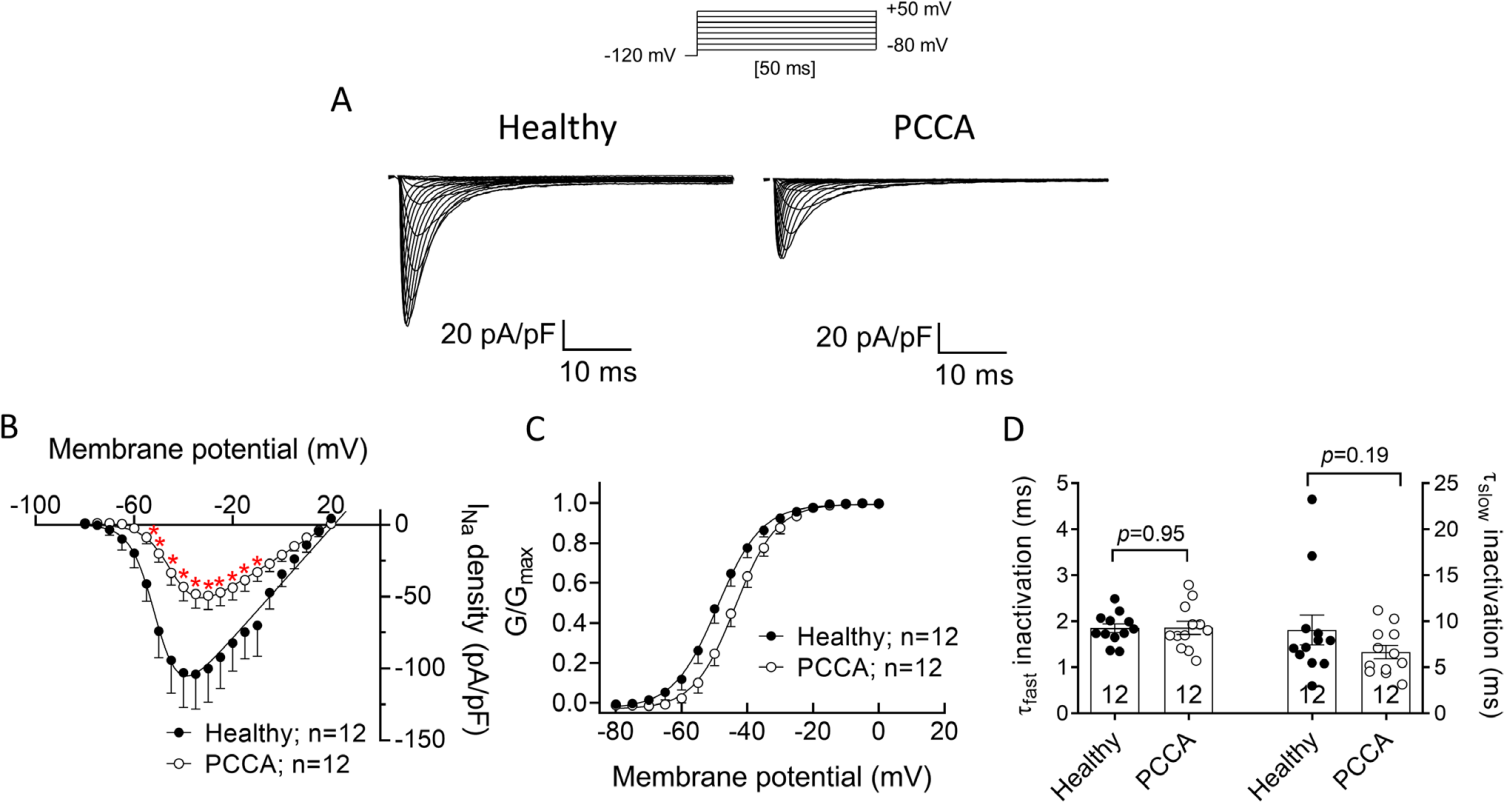
PCC deficiency reduces peak *I*_Na_. (A) Family of *I*_Na_ traces recorded by applying the pulse protocol shown at the top in a hiPSC‐CM from the healthy donor or the *PCCA*‐deficient patient. (B) Density–voltage curves of the *I*_Na_ recorded in hiPSC‐CMs from the healthy donor or the *PCCA*‐deficient patient. (C) Normalized conductance–voltage curves recorded in both experimental groups. (D) Fast (*τ*_fast_) and slow (*τ*_slow_) time constants of inactivation yielded by the biexponential fit to the decay phase of maximum peak *I*_Na_ traces. In panels B–D, each point/bar represents the mean ± SEM of the “*n*” experiments/cells indicated in each corresponding figure and in D, each dot represents one experiment. In panel B, *p < 0.05 versus healthy cells, and in panel D, the *p* values from Student's *t* comparisons are indicated.

Peak I_Na_ is one of the main determinants of cardiac excitability and conduction velocity [31]. Thus, to test whether PCC deficiency affects this current, we recorded *I*_Na_ in healthy and PCCA hiPSC‐CMs. Figure 3A shows peak *I*_Na_ traces recorded by applying the protocol shown at the top. PCCA cells displayed a significantly reduced *I*_Na_ density at potentials between −55 and +10 mV compared to healthy hiPSC‐CMs (*n* ≥ 12, *p* < 0.05) (Figure 3A,B). Moreover, the membrane potential at which the *I*_Na_ density–voltage curve peaked was depolarized, suggesting an effect on the voltage dependence of activation. Indeed, the conductance–voltage curve of the *I*_Na_ recorded in hiPSC‐CMs from the *PCCA*‐deficient patient was significantly shifted to more positive potentials compared to that of healthy cells (*V*_h_ = −43.7 ± 1.6 vs. −49.2 ± 2.0 mV, *p* < 0.05), albeit no significant differences were observed in the slope (*k* = 5.5 ± 0.4) (Figure 3C). There were also no between‐group differences in the *E*_rev_ (19.6 ± 2.8 vs. 18.6 ± 2.1 mV for healthy and PCCA cells, respectively, *p* > 0.05), demonstrating that PCC deficiency did not modify the ion selectivity of the channel. Finally, *I*_Na_ activation or inactivation kinetics were not affected either since *τ*_act_ (0.4 ± 0.03 ms), *τ*_finact_ (1.8 ± 0.1 ms) or *τ*_sinact_ (9.1 ± 1.6 ms) were not significantly modified (*n* ≥ 12, *p* > 0.05) (Figure 3D). An increase in the *I*_NaL_ can prolong APD and produce DADs [30].

**FIGURE 4:**
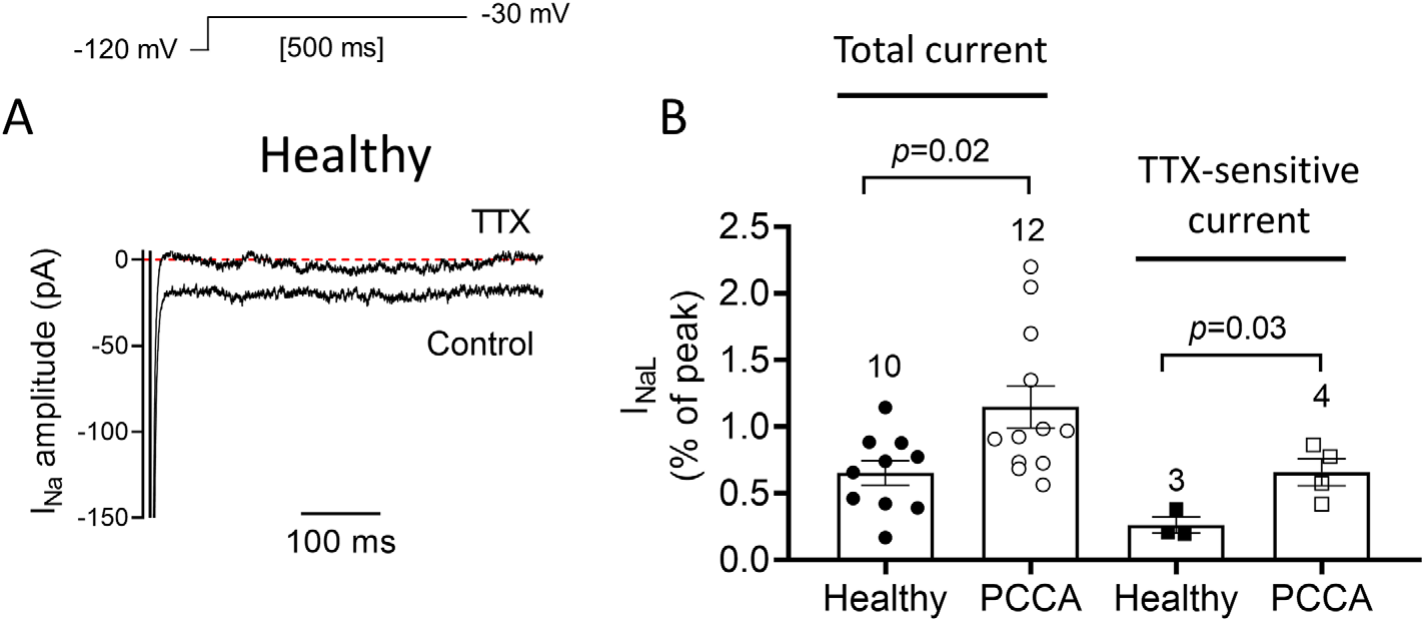
PCC deficiency increases *I*_NaL_. (A) *I*_Na_ traces recorded by applying 500‐ms pulses to −30 mV in a hiPSC‐CM from the healthy donor before and after superfusion with TTX. (B) *I*_NaL_, expressed as % of peak *I*_Na_, measured at the end of 500‐ms pulses to −30 mV (Total *I*_NaL_) and as TTX‐sensitive current in hiPSC‐CMs from the healthy donor or the *PCCA*‐deficient patient. In panel B, each bar represents the mean ± SEM of the “*n*” experiments indicated in the figure, and each dot represents one single experiment. In panel B, the *p* values from Student's *t* comparisons are indicated.

In the following group of experiments, we recorded the *I*_NaL_ in healthy and PCCA hiPSC‐CMs by applying 500‐ms pulses from −120 to −30 mV (Figure 4A,B). *I*_NaL_, measured at the end of the pulse and expressed as a percentage of peak *I*_Na_, was significantly larger in PCCA than in healthy cells (*n* ≥ 10, *p* < 0.05) (Figure 4B). To confirm these results, we also measured *I*_NaL_ as the TTX‐sensitive current (Figure 4A). The TTX‐sensitive *I*_NaL_ in PCCA hiPSC‐CMs was increased compared to that measured in cells obtained from the healthy donor (*n* ≥ 3, *p* < 0.05) (Figure 4B).

### 3.3 PCC Deficiency Decreases the *I*_CaL_ Density

**FIGURE 5:**
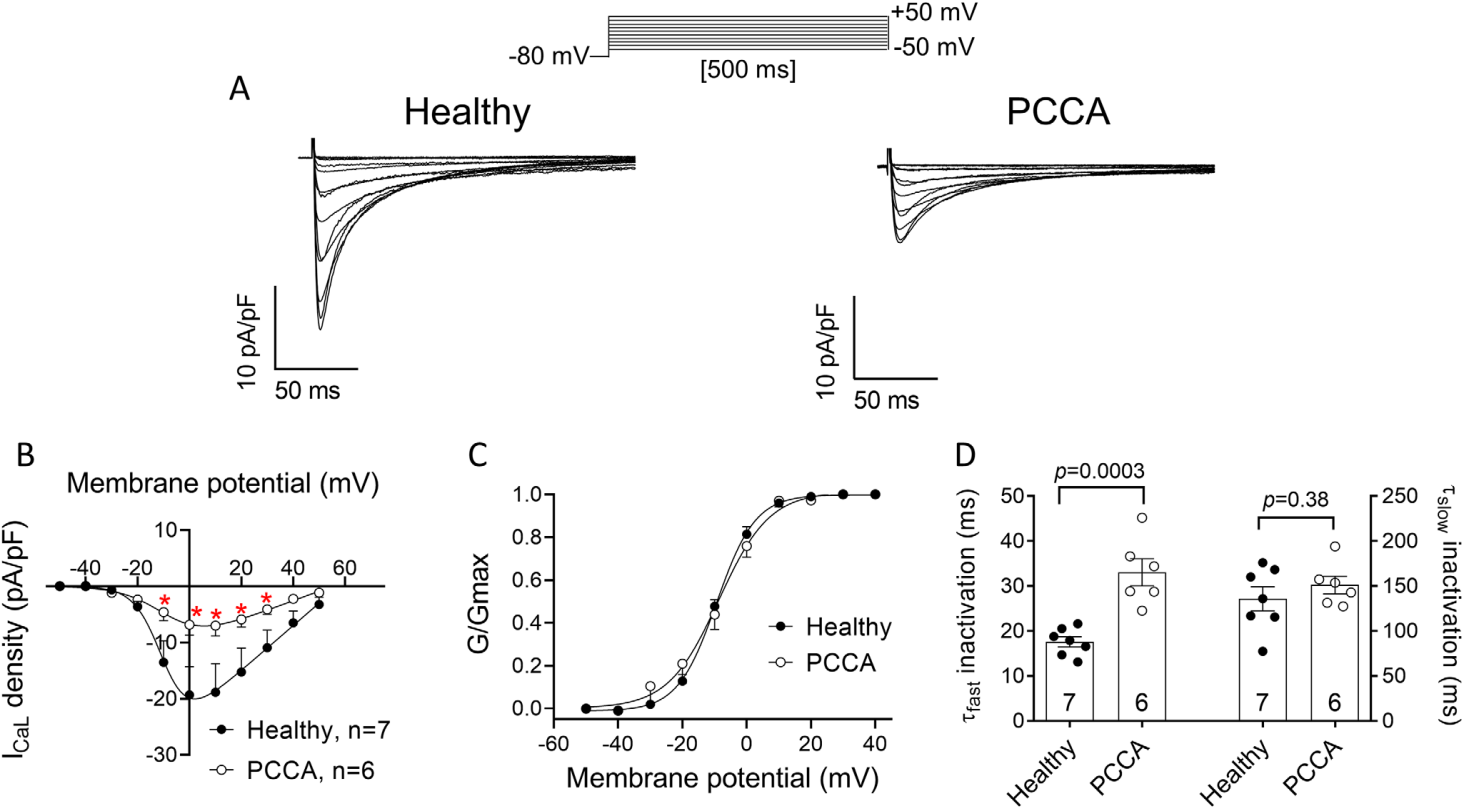
PCC deficiency decreases the *I*_CaL_ density. (A) Representative traces of *I*_CaL_ recorded in hiPSC‐CMs obtained from the healthy donor or the *PCCA*‐deficient patient by applying the pulse protocol shown at the top. Only the first 200 ms of the trace are shown for the sake of clarity. (B, C) Mean *I*_CaL_ density–voltage (B) and normalized conductance–voltage (C) curves recorded in both experimental groups. (D) Fast (*τ*_fast_) and slow (*τ*_slow_) time constants of inactivation yielded by the biexponential fit to the decay phase of *I*_CaL_ traces recorded at +10 mV. In panels B–D, each point/bar represents the mean ± SEM of 7 and 6 experiments/cells, and in panel D, each dot represents one experiment. In panel B, *p < 0.05 versus healthy cells, and in panel D, the *p* values from Student's *t* comparisons are indicated.

DADs are usually associated with alterations in intracellular Ca^2+^ handling processes leading to cytosolic and sarcoplasmic reticulum (SR) Ca^2+^ overload [30]. We first analyzed potential differences in the *I*_CaL_ recorded in healthy and PCCA hiPSC‐CMs. Figure 5A shows *I*_CaL_ traces recorded in both groups of cells by applying the protocol shown at the top. As can be observed, *I*_CaL_ density was significantly reduced at potentials between −10 and +30 mV in PCCA compared to healthy cells (*n* ≥ 6, *p* < 0.05) (Figure 5A,B). Next, we determined whether PCC deficiency induced any changes in the voltage‐ and time‐dependent properties of *I*_CaL_. Conductance–voltage curves of *I*_CaL_, constructed by plotting the normalized conductance as a function of the membrane potential, almost overlap (Figure 5C), indicating that PCC deficiency did not modify the voltage dependence of activation of the L‐type Ca^2+^ channels. Furthermore, the ion selectivity of channels was not altered either, as demonstrated by the absence of changes in the *E*_rev_ (70.1 ± 8.4 vs. 64.0 ± 4.8 mV for healthy and PCCA cells, respectively, *p* > 0.05) (Figure 5B). Regarding current kinetics, *τ*_act_ values measured in healthy and PCCA hiPSC‐CMs were very similar (1.5 ± 0.3 vs. 1.4 ± 0.1 ms; *n* ≥ 6, *p* > 0.05). However, PCC deficiency delayed the fast phase of the current decay and, thus, *τ*_finact_ values, but not *τ*_sinact_, were significantly larger in PCCA than in healthy cells (Figure 5D).

### 3.4 PCC Deficiency Increases the *I*_NCX_ Density

**FIGURE 6:**
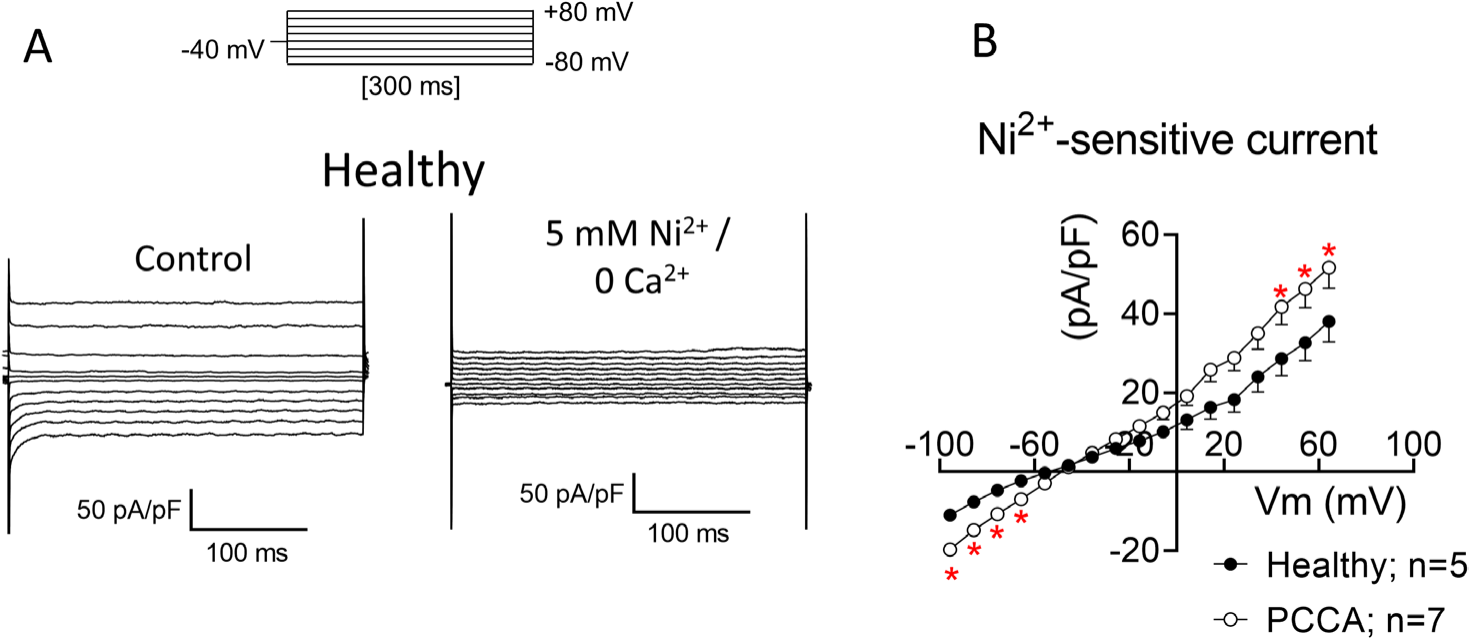
PCC deficiency increases the *I*_NCX_. (A) Representative traces of the currents recorded by applying the pulse protocol shown at the top in a hiPSC‐CM derived from the healthy donor under control conditions (left) and after superfusion with a 0 Ca^2+^ solution supplemented with 5 mM Ni^2+^ (right). (B) Density of the current sensitive to Ni^2+^ (*I*_NCX_) calculated by the subtraction of the current in the presence of Ni^2+^ from the current measured in control conditions. The curves were corrected according to the calculated LJP. In B, each point represents the mean ± SEM of the “*n*” experiments indicated in the figure. *p < 0.05 versus healthy cells.

An increase in the intracellular Na^+^ concentration caused by the augmented *I*_NaL_ can contribute to Ca^2+^ overload and may eventually increase the transient inward current (*I*_TI_) leading to DADs [30]. It is well established that *I*_NCX_ is the main responsible for *I*_TI_. Therefore, we determined whether PCC deficiency would affect *I*_NCX_ by recording it as the current sensitive to Ni^2+^, an *I*_NCX_ inhibitor [29]. Figure 6A shows representative traces of the current recorded in a healthy cardiomyocyte by applying 300‐ms pulses from a holding potential of −40 mV to potentials ranging −80 and +80 mV (top inset), first superfused with a normal extracellular solution (Control) and then with a solution containing 5 mM Ni^2+^/0 Ca^2+^. The *I*_NCX_ amplitude was measured by digitally subtracting the current recorded in the presence of Ni^2+^ from the control current (Figure 6B). As can be observed, *I*_NCX_ density was significantly increased at potentials between −80 and −50 mV (inward component) and between +60 and +80 mV (outward component) in PCCA compared to healthy hiPSC‐CMs (*n* ≥ 5, *p* < 0.05) (Figure 6B).

## 4 Discussion

Here, we have determined the electrophysiological consequences at the cardiomyocyte level of two functionally null mutations in the *PCCA* gene causing PA. Recording of APs in hiPSC‐CMs derived from the *PCCA*‐deficient patient showed a reduction in cardiomyocyte excitability, a prolongation of the APD_20_, and the appearance of DADs compared to hiPSC‐CMs differentiated from a healthy donor. Furthermore, PCC deficiency led to alterations in some individual currents compatible with the changes observed in the AP recordings, that is, the densities of *I*_NaL_ and *I*_NCX_ were augmented, while peak *I*_Na_ and *I*_CaL_ densities were markedly decreased.

Both healthy and PCCA cells generated APs with features (e.g., RMP and APD values) that reasonably resemble those of matured human ventricular cardiomyocytes [32]. A characteristic that clearly differentiates hiPSC‐CMs from real ventricular myocytes is that the former beat spontaneously, as it has been consistently demonstrated for this type of derived cardiomyocytes [27, 33, 34]. The automaticity of ventricular hiPSC‐CMs has been attributed to the smaller density of the inward rectifier current (*I*_K1_) and the greater density of the pacemaker current (*I*_f_) compared to that in genuine ventricular myocytes [33, 34]. *I*_K1_ stabilizes RMP, while I_f_ mainly determines the slope of the slow diastolic depolarization phase and thus, the firing rate of automatic cardiac cells [28, 35]. Our results show that PCC deficiency significantly reduced the spontaneous firing frequency without modifying the RMP. Thus, it is tempting to speculate that the absence of PCC activity could reduce the *I*_f_ density. However, the contribution of other alternative mechanisms cannot be ruled out (see below).

The vast majority of PCCA hiPSC‐CMs did not follow the pacing, and in those cells that followed it, the application of the stimulus took a few tens of milliseconds to trigger the AP, that is, PCC deficiency increases the latency. This fact could be the consequence of a decrease in the current responsible for the AP upstroke, which in ventricular cardiomyocytes is peak *I*_Na_. Indeed, our current recordings show that the density of peak *I*_Na_ was markedly reduced (by ≈50%) in PCCA compared to healthy cells. Moreover, the voltage dependence of *I*_Na_ activation was shifted into the depolarizing direction, suggesting that PCC deficiency modifies the biophysical properties of the channels. The *I*_Na_ decrease could explain the reduced cellular excitability that may also contribute to the lower firing frequency observed in PCCA compared to healthy hiPSC‐CMs.

On the other hand, the decrease in *I*_Na_ could be attributed to a reduction in the expression of Nav1.5 channels in the sarcolemma and/or to channel dysfunction. Although we did not investigate the underlying mechanisms responsible for the decrease in *I*_Na_, our data suggest that it is primarily due to alterations in the biophysical properties of the channels rather than a reduction in the number of channels. It has been described that there is a positive reciprocal modulation between Nav1.5 and Kir2.1 channels (the latter being responsible for *I*_K1_) such that an increase in the membrane expression of Nav1.5 channels leads to a parallel increase in the membrane expression of Kir2.1 channels, and *vice versa* [36, 37, 38]. Therefore, if PCC deficiency inhibits *I*_Na_ by reducing the number of Nav1.5 channels in the sarcolemma, it would be expected that the number of Kir2.1 channels would also decrease, thereby reducing *I*_K1_ density. We did not record *I*_K1_ in either healthy or PCCA cells; however, as already mentioned, the RMP was not significantly modified in PCCA compared to healthy hiPSC‐CMs. Since RMP depends on the density of *I*_K1_, we hypothesize that PCC deficiency does not modify *I*_K1_ and Kir2.1 channel expression at the membrane and thus does not affect Nav1.5 expression. Alternatively, it is possible that, although Kir2.1 channels may be affected, the alteration of the RMP is not apparent in individual cells due to the absence of electrotonic interactions established in cellular networks [39]. Another possible explanation could be related to the effects of accumulating propionate metabolites, which lead to mitochondrial dysfunction and may result in post‐translational oxidative modifications due to increased reactive oxygen species [18]. Supporting this, in our previous work we evaluated mitochondrial respiration in iPSC‐derived cardiomyocytes from the PCCA patient and observed significant mitochondrial dysfunction. This was evidenced by decreased oxidative phosphorylation, reduced ATP‐linked oxygen consumption, and lower maximal oxygen consumption rates. Additionally, we observed elevated levels of mitochondrial and endoplasmic reticulum stress‐related proteins, such as GRP78 and GRP75, further indicating the severity of the stress response triggered in these cells [25]. Moreover, since mitochondrial dysfunction and oxidative stress reduce peak *I*_Na_, increase *I*_NaL_, and may lead to Ca^2+^ handling alterations [40, 41, 42], it can be speculated that these mechanisms may underlie some of the PCC deficiency‐induced electrophysiological alterations described here. Additionally, the toxic metabolites may act as modifiers of the expression of genes critical to normal cardiac function mediated by miRNAs [19]. Together, these factors could specifically affect the function of the Nav1.5 channel without altering Kir2.1.

Our experiments also demonstrated that the PCCA cells displayed a larger *I*_NaL_ than healthy hiPSC‐CMs, which could explain the prolongation of the APD_20_. The *I*_NaL_ increase was confirmed by using TTX, which at micromolar concentrations preferentially inhibits *I*_NaL_ over peak *I*_Na_ in cardiac myocytes [27]. Considering the role of *I*_NaL_ in the control of ventricular APD [43], a prolongation of the duration of the whole repolarization process might be expected. Although we found a 30% prolongation of the APD_50_, this did not reach statistical significance. Nevertheless, it is possible that the *I*_CaL_ reduction observed in PCCA hiPSC‐CMs could be counteracting the prolongation of the APD induced by the *I*_NaL_ decrease.

Moreover, it has been described that an enhancement of *I*_NaL_ can lead to a Na^+^‐dependent Ca^2+^ loading of cardiomyocytes, which could eventually result in an increase in *I*_NCX_ and *I*_TI_ [43]. Interestingly, both non‐stimulated and paced PCCA hiPSC‐CMs generated DADs after APs. DADs are a form of triggered activity that carry a remarkable arrhythmogenic risk [44], traditionally linked to intracellular Ca^2+^ overload, and caused by the activation of *I*_NCX_ [43, 45]. The relationship between an increased activity of the Na^+^/Ca^2+^ exchanger and the appearance of DADs is well established in the context of atrial [46] and ventricular arrhythmias [47]. Furthermore, ventricular cardiomyocytes from a PA mouse model exhibited multiple alterations in intracellular Ca^2+^ handling leading to Ca^2+^ overload [20]. Therefore, the occurrence of DADs in *PCCA*‐deficient hiPSC‐CMs could be attributed to the concurrent increase in *I*_NaL_, *I*_NCX_, and intracellular Ca^2+^ concentration. Moreover, the *I*_CaL_ reduction observed could represent a protective mechanism against Ca^2+^ overload, as occurs in the electrical remodeling associated with chronic atrial fibrillation [48]. Further studies would be needed to identify the ultimate mechanisms underlying the observed changes in *I*_Na_, *I*_NaL_, *I*_CaL_, and *I*_NCX_ produced by PCC deficiency.

PA disease has been associated with disturbances in cardiac electrical activity, with the prolongation of the QT interval of the electrocardiogram, which can appear in up to 70% of the patients, being the most typical alteration [1, 13, 14]. In these patients, QT prolongation can be accompanied by cardiac rhythm disturbances, such as ventricular ectopic beats, life‐threatening arrhythmias, and sudden cardiac death [1, 13, 14]. The *I*_NaL_ increase described here in *PCCA* patient‐derived hiPSC‐CMs could contribute to the prolongation of the ventricular APD and QT interval, although putative alterations in outward K^+^ currents cannot be ruled out. Following this, previous research has shown that propionic acid and its metabolites (propionylcarnitine, methylcitrate) inhibit the slow component of the delayed rectifier current (*I*_Ks_) and markedly shift the voltage dependence of activation of the rapid component of the delayed rectifier current (*I*_Kr_), as demonstrated in acute experiments conducted in cell lines and hiPSC‐CMs derived from healthy donors [49]. The authors proposed that these effects could delay ventricular repolarization and, in turn, produce the QT prolongation. Strikingly, chronic exposure to these compounds did not modify the *I*_Kr_ and did not decrease, but increased the expression of Kv7.1 channels, which generate the *I*_Ks_ [49]. It is worth noting that a prolongation of APD_90_ was not apparent in isolated *PCCA‐*deficient iPSC‐CMs, probably due to the already mentioned lack of electrotonic interactions [39]. The peak *I*_Na_ reduction could decrease cardiac excitability and slow conduction velocity, and both effects may enable reentrant arrhythmias to occur. Moreover, as mentioned, DADs critically contribute to arrhythmogenesis. Importantly, our results strongly suggest that PCC deficiency may increase the risk of severe arrhythmias independently of the establishment of progressive late‐onset cardiomyopathies (e.g., DCM, HCM, or LVNC) induced by PA disease. These findings highlight the need for targeted surveillance strategies and may guide future therapeutic approaches in managing PCC deficiency.

In this context, the potential therapeutic efficacy of antiarrhythmic drugs (AADs) in modulating these effects offers a promising area for future research. Among the four classes of AADs, class I (Na^+^ channel blockers), class III (K^+^ channel blockers), and class IV (Ca^2+^ channel blockers) might not be suitable for treating arrhythmias in PA patients, as they could contribute to exacerbate systolic dysfunction, heart failure, and QT prolongation, which are observed in some PA patients. Our results demonstrating that PCC deficiency reduces peak *I*_Na_ and *I*_CaL_ and prolongs APD_20_ provide additional evidence supporting that these 3 classes of AADs cannot be used to treat arrhythmias in PA patients. Conversely, since beta blockers (class II AADs) can target intracellular Ca^2+^ handling alterations responsible for DADs, our findings suggest that these drugs may be the only class of AADs suitable for the treatment of PA‐associated arrhythmias. Remarkably, these drugs are currently the mainstay treatment for cardiac alterations in PA patients, alongside angiotensin‐converting enzyme inhibitors [1, 50]. In addition, sodium‐glucose cotransporter type 2 inhibitors (SGLT2i), such as empagliflozin and dapagliflozin, exhibit remarkable beneficial effects in patients with cardiovascular disease [51]. These drugs exert electrophysiological effects, including increasing peak *I*_Na_, inhibiting *I*_NaL_, and alleviating pathological Ca^2+^‐handling alterations [26, 52, 53, 54, 55, 56], which could counteract the electrophysiological alterations induced by PCC deficiency. Therefore, it is possible that, in the future, these drugs may play a role in managing arrhythmias associated with PA, though further studies and clinical data are necessary to confirm their efficacy.

Our study has some limitations. We have conducted the electrophysiological characterization of *PCCA*‐deficient hiPSC‐CMs, with two functionally null mutations. Previous evidence suggests that these *PCCA*‐deficient cells recapitulate most of the PA features and provide an optimal platform to model the disease [24, 25]. It would be interesting to confirm our results in other PA iPSC‐derived cardiomyocytes. To ensure that the observed changes can be attributed exclusively to PCC deficiency and not to other factors, the use of an isogenic control or the correction of the mutations via gene editing using CRISPR‐Cas9 is generally considered a useful approach. However, in the case of the mutations present in this *PCCA* patient, due to the large deletions involved, such an approach would not be feasible. In this context, it should be considered that the key features of the AP and ion current recorded in hiPSC‐CMs differentiated from the healthy donor in our study are in reasonable agreement with those of other commercial or home‐made “control” hiPSC‐CMs [26, 27, 33, 36]. This fact together with the consistency of the results strongly supports the contention that the observed differences are caused by the absence of PCC activity.

## 5 Conclusion

The characterization of the cardiac electrophysiological consequences of *PCCA* gene defects causing PA demonstrated that the disease per se produces proarrhythmic AP and ion current alterations at the cellular level, beyond those secondary to the development of PA‐induced cardiomyopathy in the individual. Our findings not only provide new insights into the electrophysiological mechanisms underlying the cardiac complications of PA but also underscore the relevance of the hiPSC‐CM model as a valuable tool for investigating the disease and for testing the effectiveness of existing and emerging therapies in a controlled, patient‐specific manner with the final aim of mitigating the arrhythmogenic risk and improving the cardiac prognosis in PA patients.

## Author Contributions

**Anabel Cámara‐Checa:** visualization, methodology, investigation, formal analysis. **Mar Álvarez:** visualization, methodology, investigation, formal analysis. **Josu Rapún:** visualization, methodology, investigation, formal analysis. **Sara Pérez‐Martín:** visualization, methodology, investigation, formal analysis. **Roberto Núñez‐Fernández:** visualization, methodology, investigation, formal analysis. **Marcos Rubio‐Alarcón:** visualization, methodology, investigation, formal analysis. **Teresa Crespo‐García:** visualization, methodology, investigation, formal analysis. **Lourdes R. Desviat:** writing – review and editing, resources, funding acquisition, conceptualization. **Eva Delpón:** writing – review and editing, supervision, resources, data curation, methodology, funding acquisition, conceptualization. **Ricardo Caballero:** writing – review and editing, writing – original draft, supervision, resources, data curation, methodology, funding acquisition, conceptualization. **Eva Richard:** writing – review and editing, writing – original draft, supervision, methodology, resources, investigation, funding acquisition, conceptualization. Ricardo Caballero and Eva Richard are the guarantors for the article.

## Ethics Statement

The study was approved by the Ethics Committee of the Autonomous University of Madrid (project identification code CEI‐134‐2830; date of approval: November 3, 2023) and by the authorization of “Dirección General de Investigación Sanitaria y Documentación”, Community of Madrid (data of approval: February, 19 2024). This article does not contain any studies with animal subjects performed by any of the authors.

## Consent

All procedures followed were in accordance with the ethical standards of the responsible committee on human experimentation (institutional and national), and with the Helsinki Declaration of 1975, as revised in 2000 (5). Informed consent was obtained from the patient's family for being included in the study. No identifying patient information is included in this article.

## Conflicts of Interest

The authors declare no conflicts of interest.

## Data Availability

Data will be made available on request.
